# Assessment of Paclitaxel Drug-Coated Balloon-Only Angioplasty for Multivessel Coronary Artery Disease

**DOI:** 10.3390/jcm15010204

**Published:** 2025-12-26

**Authors:** Ioannis Merinopoulos, Natasha Corballis, U Bhalraam, Rajkumar Natarajan, Tharusha Gunawardena, Johannes Reinhold, Clint Maart, Chris Sawh, Sreekumar Sulfi, Trevor Wistow, Alisdair Ryding, Tim Gilbert, Vassilios S. Vassiliou, Simon C. Eccleshall

**Affiliations:** 1 Department of Cardiology, Norfolk and Norwich University Hospital, Colney Lane, Norwich NR4 7UY, UK; natasha.corballis@nnuh.nhs.uk (N.C.); u.bhalraam@nnuh.nhs.uk (U.B.); rajkumar.natarajan@nnuh.nhs.uk (R.N.); johannes.reinhold@nnuh.nhs.uk (J.R.); clint.maart@nnuh.nhs.uk (C.M.); chris.sawh@nnuh.nhs.uk (C.S.); sreekumar.sulfi@nnuh.nhs.uk (S.S.); trevor.wistow@nnuh.nhs.uk (T.W.); alisdair.ryding@nnuh.nhs.uk (A.R.); timothy.gilbert@nnuh.nhs.uk (T.G.); v.vassiliou@uea.ac.uk (V.S.V.); 2 Norwich Medical School, University of East Anglia, Research Park, Norwich NR4 7TJ, UK

**Keywords:** multivessel angioplasty, drug coated balloon only angioplasty, drug eluting stent

## Abstract

**Background**: There are limited data about the use of drug-coated balloon (DCB)-only angioplasty for multivessel coronary disease. **Objectives**: The aim of this study was to assess the safety and efficacy of DCB-only angioplasty as compared with second-generation drug-eluting stents (DESs) in patients undergoing multivessel angioplasty. **Methods**: We compared major adverse cardiovascular events (MACEs) in all patients undergoing multivessel angioplasty in our institution from 1 January 2015 until 15 November 2019, with either the DCB-only or DES-only strategy. The primary endpoint of our study was a MACE, including cardiovascular mortality, acute coronary syndrome, target lesion revascularisation, stroke, or major bleeding. Data were analysed using Cox regression models, Kaplan–Meier estimator plots, and propensity score matching. **Results**: A total of 159 consecutive patients treated with DCB-only angioplasty and 222 consecutive patients treated with DES-only angioplasty were identified. The majority of the vessels treated were large vessels (>3 mm). After a median follow-up of 4 years, a total of 52 (33%) patients in the DCB and 73 (33%) patients in the DES group encountered a MACE (*p* = 0.97). The results did not change following propensity score matching. On multivariate Cox regression analysis in the propensity score-matched cohort, atrial fibrillation [HR = 2.37, CI: (1.22–4.61), *p* = 0.011] and diabetes [HR = 1.71, CI: (1.13–2.60), *p* = 0.011] were the only independent adverse predictors of MACE. **Conclusions**: DCB-only angioplasty appears to be safe and efficient when compared to DES for multivessel angioplasty in terms of MACEs after a follow-up of 4 years.

## 1. Introduction

Percutaneous coronary intervention (PCI) has progressed from balloon angioplasty to bare metal stent and then multiple generations of drug-eluting stents (DESs). Stents were initially developed to alleviate the limitations of balloon angioplasty (vessel-threatening dissections and vessel recoil) and have shown to improve long-term patient outcomes [1]. Despite the improved patient outcomes, very late stent-related adverse events continue to occur at a rate of about 2% per year with no plateau effect [2]. The length and number of stents are associated with worse patient outcomes even in the era of second-generation DES [3]. Stenting longer than 40 mm is associated with target lesion failure and stent thrombosis [3]. A drug-coated balloon (DCB)-based approach has been proposed in the literature as a treatment strategy for complex coronary anatomy aiming to simplify complex stenting [4].

DCB-only angioplasty is a treatment strategy which utilises the concept of ‘leave nothing behind’ and has received increasing interest more recently. It allows for the delivery of an anti-restenotic agent to the vessel wall without implantation of a permanent metallic scaffold [5]. The absence of a stent caging the vessel allows for better preservation of arterial vasomotion while simultaneously mitigating the stent-related mechanisms leading to stent thrombosis, in-stent restenosis, and accelerated atherosclerosis [6]. Patients with multivessel disease undergoing multivessel angioplasty might require excessive length of stents and be at increased risk of stent-related complications. Shin et al. were the first to demonstrate that reducing the length of stents by a hybrid DCB-based approach (DCB-only or DCB combined with DES) is associated with better patient outcomes in the context of multivessel PCI [7]. The present study aims to assess the safety and efficacy of a DCB-only approach compared to DES-only in patients undergoing multivessel PCI.

## 2. Methods

The assessment of paclitaxel drug-coated balloon-only angioplasty for multivessel disease is an investigator-initiated, retrospective, propensity-matched, single-centre cohort study of the SPARTAN Norwich Registry. The decision for DCB or DES was left to the discretion of the operator. Our institution prospectively enrols all patients undergoing PCI into a clinical database with baseline patient and angiographic characteristics. Our study was approved by the Northwest Haydock (17/NW/0278) UK research ethics committee. Given the retrospective nature of our study, the confidentiality advisory group waived the need for consent (17/CAG/0145). We included all patients undergoing multivessel PCI in the same procedure from 1 January 2015 until 15 November 2019 as this was the final day that we could include patients according to the ethics committee. We excluded patients with cardiac arrest, intubation, or cardiogenic shock as the outcomes of these patients were significantly influenced by their severe clinical presentation rather than the interventional treatment strategy. Electronic hospital records were reviewed if required to supplement the clinical data of our database. All angiograms were reviewed by an operator (NC) blinded to the patient outcomes to confirm the accuracy of the angiographic data and treatment strategy. Calcification, tortuosity, and diffuse disease were assessed angiographically. The largest pre/post-dilatation balloon, for DCB or DES, was considered as the vessel diameter and the DCB or DES length was considered as the lesion length.

The primary endpoint was major adverse cardiovascular events (MACEs) including cardiovascular death, acute coronary syndrome (ACS), target lesion revascularisation (TLR), stroke, or major bleeding. We utilised Hospital Episodes Statistics (HES) from the NHS in England to obtain patient outcomes. HES is a national database containing details of all admissions, outpatient appointments, and accident and emergency attendances at any NHS hospital in England. The ICD-10 diagnostic codes provided in Supplementary Table S1 were used to extract patients’ outcomes. Three blinded adjudicators classified all deaths as cardiovascular or non-cardiovascular according to the academic research consortium 2 consensus [8].

R statistical software (version 4.0 or later) was used for the statistical analysis. The Wilcoxon rank sum test was used to compare continuous variables, which are presented as the median with interquartile range (IQR). The Pearson’s Chi-squared test or Fisher’s exact test, where appropriate, were used to compare categorical variables, which are presented as frequencies with percentages. Baseline characteristics were summarised using descriptive statistics and compared between treatment groups.

Cox proportional hazards regression models were used to perform survival analyses. A univariate Cox regression analysis was conducted for all clinical and angiographic variables to identify potential predictors of major adverse cardiovascular events (MACEs) and individual endpoints. Variables with clinical relevance were included in multivariable Cox regression models using stepwise selection (bidirectional elimination) to identify independent predictors of outcomes. Hazard ratios (HRs) with 95% confidence intervals (CIs) were calculated, with results exponentiated for interpretation.

To account for potential confounding and selection bias, propensity score matching was performed using optimal matching with a 1:1 ratio. Despite relatively balanced baseline characteristics, propensity score matching was employed to enhance comparability and provide more robust estimates. The propensity score model was pre-specified and included clinically relevant variables known to influence both treatment selection and outcomes: age, sex, past medical history of coronary artery bypass grafting (CABG), angina, atrial fibrillation, diabetes mellitus, and glomerular filtration rate (GFR).

These variables were selected based on their established prognostic importance in coronary intervention outcomes. Balance between matched groups was assessed using standardised differences, with differences >0.1 considered meaningful imbalances, and graphical methods.

Kaplan–Meier survival curves were constructed to visualise the cumulative hazard for MACEs, with differences between groups assessed using the log-rank test. Time-to-event was calculated from the index procedure date to the first occurrence of the endpoint or to the last follow-up date (31 March 2021). All statistical analyses were two-sided, with statistical significance defined as *p* < 0.05.

## 3. Results

A total of 159 consecutive patients treated with DCB-only angioplasty and 222 consecutive patients treated with DES-only angioplasty were identified. The median age was 72 (IQR: 60–78) and 70 (IQR: 61–76) years old for the DCB and DES groups, respectively. Male patients accounted for 82% and 77% of the DCB and DES groups, respectively. Table 1 shows that the groups were well balanced in terms of the baseline clinical characteristics. The only difference was that the DCB group had a significantly higher proportion of patients with previous PCI (33% vs. 22%, *p* = 0.011). They were also well balanced in terms of the angiographic characteristics. However, the DCB group had a significantly higher proportion of patients with diffuse disease (33% vs. 24%, *p* = 0.042) and tortuosity (23% vs. 10%, *p* = 0.001). The DCB group had a significantly smaller median vessel diameter [3.00 (IQR: 2.75–3.25) vs. 3.25 (IQR: 3.00–3.50), *p* < 0.001].

The median follow-up was 3.9 (IQR: 2.5–5.0) years for the whole cohort, 4.0 (IQR: 2.6–5.0) for the DCB group, and 3.8 (IQR: 2.5–5.1) years for the DES group (*p* = 0.75). A total of 52 (33%) patients in the DCB group and 73 (33%) patients in the DES group encountered MACEs (*p* = 0.97). The great majority of the DCBs used were SeQuent Please or SeQuent Please NEO (97.3%), with a small percentage being Falcon (2.7%). The drug-eluting stent platforms used were Promus Premier (38.5%), Synergy (34.5%), Onyx (19.4%), and Xience (7.6%).

**Figure 1:**
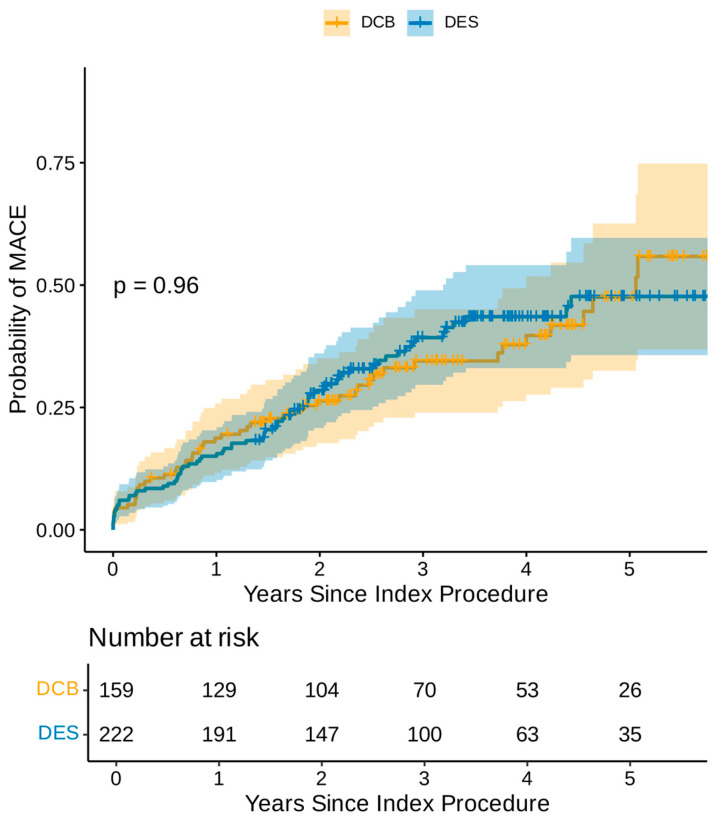
Kaplan–Meier estimator plot of MACE for DCB-only vs. DES-only angioplasty (non-propensity matched).

Following the univariable Cox regression analysis, left main stem PCI, heavy calcification and a history of atrial fibrillation, diabetes, and coronary artery bypass grafts were found to be adverse predictors of MACEs, while elective PCI and family history of ischaemic heart disease were favourable prognostic indicators (Supplementary Table S2). The multivariate Cox regression analysis demonstrated that atrial fibrillation, diabetes, and left main stem treatment were the only independent adverse prognostic indicators for MACEs, while family history of ischaemic heart disease was the only independent favourable prognostic indicator (Table 2). The Kaplan–Meier estimator plot demonstrated no difference in overall MACEs between DCB and DES, as demonstrated in Figure 1.

**Figure 2:**
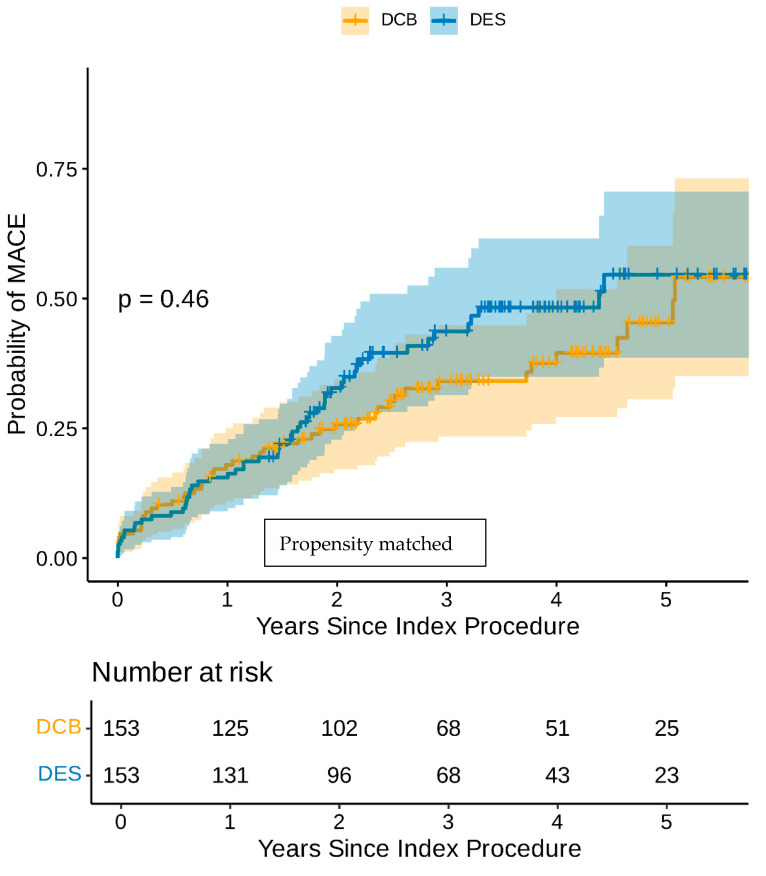
Kaplan–Meier estimator plot of MACE for DCB-only vs. DES-only angioplasty (propensity-matched).

Even though the groups were well balanced with regard to the baseline clinical and angiographic characteristics, a propensity score-matched analysis was undertaken. The baseline characteristics of the propensity score-matched cohort are shown in Table 3. The univariate Cox regression analysis in the propensity score-matched cohort demonstrated that CABG, AF, diabetes, and heavy calcification were poor prognostic indicators, while family history of IHD was the only good prognostic indicator (Supplementary Table S3). The multivariate Cox regression analysis in the propensity-matched cohort identified AF and diabetes as the only independent poor prognostic indicators for MACEs (Table 4). The Kaplan–Meier estimator plot demonstrated that there was no difference between the DCB-only and DES-only angioplasty in terms of MACEs (Figure 2). Furthermore, there was no difference in any of the individual components of MACEs between the DCB-only or DES groups even though there were consistently less adverse events in the DCB-only group (Table 5).

## 4. Discussion

This is the first and largest cohort analysis assessing a DCB-only strategy compared with second-generation DES-only strategy for multivessel de novo PCI in a single procedure, demonstrating no difference in overall MACEs after 4 years of follow-up.

Even though second-generation DESs have significantly improved the outcomes of PCI, there remains a long-term risk of stent-related adverse events [2]. Very late stent-related ischaemic events continue to occur at a rate of approximately 2% per year, irrespective of stent type and with no evidence of a plateau effect up to 5 years [2]. Neoatherosclerosis, uncovered struts, malposition, and stent under-expansion are some of the mechanisms leading to stent failures, while overlapping stents and increasing stent lengths are important stent-related factors leading to worse outcomes [9]. A stent length of more than 40 mm is associated with less favourable clinical outcomes such as target lesion failure and stent thrombosis even in the contemporary era of second-generation DES [3]. Over the years, efforts have primarily focused on improving the stent technology, intensifying pharmacotherapy, and optimising the stent implantation with intravascular imaging in order to reduce the long-term stent-related complications [10,11]. Even though this approach is necessary, a different strategy, using metal-free angioplasty, could also be useful in further reducing stent-related complications [12]. DCB angioplasty is based on the concept of ‘leaving nothing behind’, utilising a balloon platform to deliver an anti-proliferative drug to the vessel wall. Initially, paclitaxel was the preferred drug due to its favourable lipophilicity; however, more recently, sirolimus DCBs have also been developed [6]. Irrespective of the drug used, the principle is that the anti-restenotic agent will reduce the development of intimal hyperplasia following the vessel wall injury from the balloon angioplasty while the absence of metal will mitigate long-term stent-related adverse events such as neoatherosclerosis [6].

Over the last few years, there has been an increasing interest in DCB angioplasty for de novo disease, either alone or as a hybrid strategy combined with DES. DCBs continue to be evaluated in a number of clinical (stable angina, acute coronary syndrome, and high bleeding risk) or anatomical settings (small vessel disease, large vessels, diffuse disease, and bifurcations) with variable but overall encouraging results [6,13,14,15,16]. Indeed, a DCB-based approach has been previously proposed as a strategy for approaching complicated coronary anatomy to reduce the length of stents and simplify complex stenting [4]. Multivessel disease is a particularly challenging clinical scenario, where coronary artery bypass is beneficial to multiple stenting, especially regarding intermediate or high SYNATX scores [17]. Given that multiple, long stents are associated with worse long-term patient outcomes, multivessel PCI is a clinical scenario where metal-free PCI could demonstrate its potential benefit. In that context, Shin et al. was able to demonstrate that a DCB-based treatment had significantly lower MACEs when compared to DES-only treatments [7]. Shin et al. compared 254 patients undergoing multivessel PCI with either DCB-only or DCB and DES (hybrid) approaches against 254 patients treated with a DES-only strategy selected from the PTRG-DES (Platelet Function and Genotype-Related Long-Term Prognosis in Drug-Eluting Stent-Treated Patients with Coronary Artery Disease) consortium. The hybrid approach was utilised in about two-thirds of the patients in the DCB-based group while the remaining one-third had DCB-only angioplasty, and small vessels were mainly treated with DCB (<3 mm) [7]. Nevertheless, the study demonstrated that reducing the burden of stents in patients undergoing multivessel PCI significantly improved patient outcomes at 2 years. Furthermore, it also demonstrated a plateau effect in the DCB-based group after 1 year [7].

Our study is the first and largest analysis comparing DCB-only angioplasty with DES-only angioplasty for multivessel PCI. We have shown that DCB-only angioplasty for multivessel PCI is safe and efficient with no difference in overall MACEs compared to DES-only angioplasty after a 4-year follow-up period. Furthermore, the results were similar following propensity matching. We recruited consecutive patients and, importantly, large vessels were treated in both groups. The DCB-only group had significantly more patients with diffuse disease, tortuous vessels, and previous PCI, indicating more anatomically complex coronary disease. These differences could be explained by the fact that our institution has vast experience with DCB-only angioplasty, which might be the preferred strategy for complex anatomical scenarios such as diffuse disease. The differences remained even after propensity score matching.

Our study builds on the evidence presented by Shin et al., which had demonstrated that a DCB-based treatment for multivessel PCI may be used either alone for smaller vessels or as a hybrid approach in combination with DES for larger vessels [7]. We did not demonstrate a statistical benefit of DCB-only angioplasty compared to DES, but the overall MACEs and all its individual components were numerically improved in the DCB group, suggesting a consistent trend. Our study supports the use of DCB-only angioplasty for multivessel disease in large vessels, including diffuse disease or tortuous vessels, and provides evidence of favourable results at a median follow-up of 4 years. Randomised clinical trials are warranted to assess the effects of metal-free PCI for multivessel disease.

### Study Limitations

The retrospective nature of our study is a possible source of bias, suggesting that our results should be approached with caution; however, we included all consecutive patients meeting our inclusion criteria, trying to ameliorate referral bias. All cases were recruited from a single centre with vast experience in DCB-only angioplasty; therefore, the results might not be reproducible to centres with less experience. The lack of a core-lab analysis and low usage of intracoronary imaging are further limitations of our study. The decision to use DCB or DES was left to the discretion of the operator, which is a possible source of bias as the decision was not randomised. However, there were only a few differences in the baseline characteristics between the groups (in favour of DES) and we did use propensity score matching analysis to confirm that our results are robust.

## 5. Conclusions

This is the first and largest cohort analysis comparing DCB-only angioplasty versus second-generation DES-only angioplasty for multivessel PCI. We have demonstrated no evidence of differences in MACEs after a long-term follow-up period of 4 years, even after propensity score matching.

## Figures and Tables

**Table 1:** Baseline clinical and angiographic characteristics.

Characteristic	Overall N = 381	DCB N = 159	DES N = 222	*p*-Value
Age (years), Median (IQR)	70 (61–78)	72 (60–78)	70 (61–76)	0.29 ^1^
Male, n (%)	302 (79)	131 (82)	171 (77)	0.20 ^2^
Presentation, n (%)				0.29 ^2^
STEMI	48 (13)	15 (9.4)	33 (15)	
NSTEMI	150 (39)	65 (41)	85 (38)	
Elective	183 (48)	79 (50)	104 (47)	
PCI Strategy, n (%)				**<0.001** ^2^
DCB-Only	159 (42)	159 (100)	0 (0)	
DES	222 (58)	0 (0)	222 (100)	
Hypercholesteraemia, n (%)	101 (27)	42 (26)	59 (27)	0.97 ^2^
Hypertension, n (%)	198 (52)	83 (52)	115 (52)	0.94 ^2^
PVD, n (%)	19 (5.0)	9 (5.7)	10 (4.5)	0.61 ^2^
Stroke, n (%)	19 (5.0)	12 (7.5)	7 (3.2)	0.052 ^2^
Myocardial Infarction, n (%)	101 (27)	50 (31)	51 (23)	0.065 ^2^
PCI, n (%)	101 (27)	53 (33)	48 (22)	**0.011** ^2^
CABG, n (%)	29 (7.6)	16 (10)	13 (5.9)	0.13 ^2^
Heart Failure, n (%)	13 (3.4)	8 (5.0)	5 (2.3)	0.14 ^2^
Family History of IHD, n (%)	64 (17)	32 (20)	32 (14)	0.14 ^2^
Atrial Fibrillation, n (%)	18 (4.7)	10 (6.3)	8 (3.6)	0.22 ^2^
COPD, n (%)	24 (6.3)	9 (5.7)	15 (6.8)	0.66 ^2^
Diabetes, n (%)	85 (22)	35 (22)	50 (23)	0.91 ^2^
Smoking Status, n (%)				0.74 ^2^
Never Smoked	138 (38)	60 (38)	78 (37)	
Current/Ex-Smoker	230 (63)	96 (62)	134 (63)	
Unknown	13	3	10	
GFR, Median (IQR)	80 (63–98)	79 (60–97)	81 (66–99)	0.23 ^1^
Left Main Stem	67 (18)	23 (14)	44 (20)	0.18 ^2^
Heavy Calcification, n (%)	118 (31)	56 (35)	62 (28)	0.13 ^2^
Diffuse Disease, n (%)	106 (28)	53 (33)	53 (24)	**0.042** ^2^
Tortuosity, n (%)	59 (15)	36 (23)	23 (10)	**0.001** ^2^
Vessel Diameter, Median (IQR)	3.17 (2.88–3.50)	3.00 (2.75–3.25)	3.25 (3.00–3.50)	**<0.001** ^1^
Lesion Length, Median (IQR)	50 (40–67)	50 (40–60)	54 (38–70)	0.51 ^1^

^1^ Wilcoxon rank sum test; ^2^ Pearson’s Chi-squared test. Baseline clinical and angiographic characteristics of patients undergoing multivessel PCI with DCB-only or DES-only angioplasty. Abbreviations: DCB: drug-coated balloon, DES: drug-eluting stent, STEMI: ST elevation myocardial infarction, NSTEMI: non-ST elevation myocardial infarction, PVD: peripheral vascular disease, PCI: percutaneous coronary intervention, IHD: ischaemic heart disease, COPD: chronic obstructive pulmonary disease, and GFR: glomerular filtration rate. Bold simply indicates significant *p* value.

**Table 2:** Multivariate Cox regression analysis.

MACE (Multivariate)	N	HR (95% CI) ^1^	*p*-Value
Drug-Eluting Stent	365	0.99 (0.69 to 1.43)	0.96
Left Main Stem	365	1.61 (1.04 to 2.48)	**0.032**
Atrial Fibrillation	365	2.23 (1.15 to 4.31)	**0.018**
Diabetes Mellitus	365	1.73 (1.18 to 2.55)	**0.005**
Family History of Coronary Artery Disease	365	0.50 (0.28 to 0.89)	**0.018**

^1^ HR = Hazard Ratio, CI = Confidence Interval. Multivariate Cox regression analysis for major adverse cardiovascular events. Bold simply indicates significant *p* value.

**Table 3:** Baseline characteristics for the propensity score-matched cohort.

Characteristic	Overall N = 306	DCB N = 153	DES N = 153	*p*-Value
Age, Median (IQR)	71 (61–78)	72 (60–78)	70 (62–78)	0.72 ^1^
Male, n (%)	249 (81)	125 (82)	124 (81)	0.88 ^2^
Presentation, n (%)				0.19 ^2^
STEMI	36 (12)	13 (8.5)	23 (15)	
NSTEMI	123 (40)	62 (41)	61 (40)	
Elective	147 (48)	78 (51)	69 (45)	
LMS PCI n (%)	54 (18)	20 (13)	34 (22)	**0.036** ^2^
PCI Strategy, n (%)				**<0.001** ^3^
DCB	153 (50)	153 (100)	0 (0)	
DES	153 (50)	0 (0)	153 (100)	
Hypercholesterolaemia, n (%)	74 (24)	41 (27)	33 (22)	0.29 ^2^
Hypertension, n (%)	156 (51)	79 (52)	77 (50)	0.82 ^2^
PVD, n (%)	18 (5.9)	9 (5.9)	9 (5.9)	>0.99 ^2^
Stroke, n (%)	18 (5.9)	12 (7.8)	6 (3.9)	0.14 ^2^
Myocardial Infarction, n (%)	79 (26)	47 (31)	32 (21)	0.050 ^2^
Previous PCI, n (%)	82 (27)	51 (33)	31 (20)	**0.010** ^2^
CABG, n (%)	29 (9.5)	16 (10)	13 (8.5)	0.56 ^2^
Heart Failure, n (%)	11 (3.6)	7 (4.6)	4 (2.6)	0.36 ^2^
Angina, n (%)	58 (19)	27 (18)	31 (20)	0.56 ^2^
Family History of IHD, n (%)	53 (17)	32 (21)	21 (14)	0.10 ^2^
Atrial Fibrillation, n (%)	18 (5.9)	10 (6.5)	8 (5.2)	0.63 ^2^
COPD, n (%)	18 (5.9)	9 (5.9)	9 (5.9)	>0.99 ^2^
Diabetes, n (%)	67 (22)	32 (21)	35 (23)	0.68 ^2^
Smoking Status, n (%)				0.40 ^2^
Never Smoked	109 (36)	58 (38)	51 (33)	
Current/Ex-Smoker	197 (64)	95 (62)	102 (67)	
GFR, Median (IQR)	79 (62–96)	78 (60–97)	79 (63–94)	0.87 ^1^
Heavy Calcification, n (%)	100 (33)	54 (35)	46 (30)	0.33 ^2^
Diffuse Disease, n (%)	90 (29)	53 (35)	37 (24)	**0.045** ^2^
Tortuosity, n (%)	53 (17)	34 (22)	19 (12)	**0.023** ^2^
Vessel Diameter, Median (IQR)	3.13 (2.75–3.50)	3.00 (2.75–3.25)	3.25 (3.00–3.63)	**<0.001** ^1^
Lesion Length, Median (IQR)	50 (40–69)	50 (40–60)	53 (38–72)	0.64 ^1^

^1^ Wilcoxon rank sum test; ^2^ Pearson’s Chi-squared test; ^3^ Fisher’s exact test. Baseline clinical and angiographic characteristics of patients undergoing multivessel PCI with DCB-only or DES-only angioplasty. DCB: drug-coated balloon, DES: drug-eluting stent, STEMI: ST elevation myocardial infarction, NSTEMI: non-ST elevation myocardial infarction, PVD: peripheral vascular disease, PCI: percutaneous coronary intervention, IHD: ischaemic heart disease, COPD: chronic obstructive pulmonary disease, GFR: glomerular filtration rate, and LMS: left main stem. Bold simply indicates that the *p* value is significant.

**Table 4:** Multivariate Cox regression analysis for the propensity score-matched cohort.

MACE (Multivariate)	N	HR (95% CI)	*p*-Value
DES	306	1.11 (0.76 to 1.64)	0.59
CABG	306	1.62 (0.93 to 2.82)	0.086
Atrial Fibrillation	306	2.37 (1.22 to 4.61)	**0.011**
Diabetes Mellitus	306	1.71 (1.13 to 2.60)	**0.011**
Family History of Coronary Artery Disease	306	0.55 (0.30 to 1.02)	0.057

Abbreviations: CI = confidence interval, HR = hazard ratio, MACE: major adverse cardiovascular event, DES: drug-eluting stent, and CABG: coronary artery bypass graft. Bold simply indicates that the *p* value is significant.

**Table 5:** Breakdown of the individual MACE components in the propensity score-matched cohort.

	DCB (n = 153), n (%)	DES (n = 153), n (%)	*p*-Value
Cardiovascular death	9 (5.9)	11 (7.2)	0.65
Acute coronary syndrome	19 (12.4)	29 (19.0)	0.12
Target lesion revascularisation	12 (7.8)	13 (8.5)	0.83
Stroke	5 (3.3)	6 (3.9)	0.75
Major bleeding	11 (7.2)	13 (8.5)	0.68
Any MACE	48 (31.4)	56 (36.6)	0.36

Abbreviations: DCB = drug-coated balloon, DES = drug-eluting stent, and MACE = major adverse cardiovascular events.

## Data Availability

Data can be made available following appropriate request to the corresponding author.

## Supplementary Materials

The following supporting information can be downloaded at: https://www.mdpi.com/article/10.3390/jcm15010204/s1.

## References

[1] Yerasi C., Case B.C., Forrestal B.J., Torguson R., Weintraub W.S., Garcia-Garcia H.M., Waksman R. (2020). Drug-Coated Balloon for De Novo Coronary Artery Disease. J. Am. Coll. Cardiol..

[2] Madhavan M.V., Kirtane A.J., Redfors B., Généreux P., Ben-Yehuda O., Palmerini T., Benedetto U., Biondi-Zoccai G., Smits P.C., von Birgelen C. (2020). Stent-Related Adverse Events >1 Year After Percutaneous Coronary Intervention. J. Am. Coll. Cardiol..

[3] Kong M.G., Han J.-K., Kang J.-H., Zheng C., Yang H.-M., Park K.W., Kang H.-J., Koo B.-K., Chae I.-H., Kim H.-S. (2021). Clinical outcomes of long stenting in the drug-eluting stent era: Patient-level pooled analysis from the GRAND-DES registry. EuroIntervention.

[4] Chaddad R., El-Mokdad R., Lazar L., Cortese B. (2022). DCBs as an adjuvant tool to DES for very complex coronary lesions. Rev. Cardiovasc. Med..

[5] Jeger R.V., Eccleshall S., Wan Ahmad W.A., Ge J., Poerner T.C., Shin E.S., Alfonso F., Latib A., Ong P.J., Rissanen T.T. (2020). Drug-Coated Balloons for Coronary Artery Disease: Third Report of the International DCB Consensus Group. JACC Cardiovasc. Interv..

[6] Giacoppo D., Saucedo J., Scheller B. (2023). Coronary Drug-Coated Balloons for De Novo and In-Stent Restenosis Indications. J. Soc. Cardiovasc. Angiogr. Interv..

[7] Shin E.-S., Jun E.J., Kim S., Kim B., Kim T.-H., Sohn C.-B., Her A.-Y., Park Y., Cho J.R., Jeong Y.-H. (2023). Clinical Impact of Drug-Coated Balloon-Based Percutaneous Coronary Intervention in Patients With Multivessel Coronary Artery Disease. JACC Cardiovasc. Interv..

[8] Garcia-Garcia H.M., McFadden E.P., Farb A., Mehran R., Stone G.W., Spertus J., Onuma Y., Morel M.A., Van Es G.A., Zuckerman B. (2018). Standardized end point definitions for coronary intervention trials: The academic research consortium-2 consensus document. Circulation.

[9] Stefanini G.G., Alfonso F., Barbato E., Byrne R., Capodanno D., Colleran R., Escaned J., Giacoppo D., Kunadian V., Lansky A. (2020). Management of Myocardial Revascularization Failure: An Expert Consensus Document of the EAPCI. EuroIntervention.

[10] Gao X.-F., Ge Z., Kong X.-Q., Kan J., Han L., Lu S., Tian N.-L., Lin S., Lu Q.-H., Wang X.-Y. (2021). 3-Year Outcomes of the ULTIMATE Trial Comparing Intravascular Ultrasound Versus Angiography-Guided Drug-Eluting Stent Implantation. JACC Cardiovasc. Interv..

[11] Kereiakes D.J., Windecker S., Jobe R.L., Mehta S.R., Sarembock I.J., Feldman R.L., Stein B., Dubois C., Grady T., Saito S. (2019). Clinical Outcomes Following Implantation of Thin-Strut, Bioabsorbable Polymer-Coated, Everolimus-Eluting SYNERGY Stents: Final 5-Year Results of the EVOLVE II Randomized Trial. Circ. Cardiovasc. Interv..

[12] Eccleshall S., Scheller B. (2024). Less metal—The latest evolution in PCI. AsiaIntervention.

[13] Merinopoulos I., Gunawardena T., Corballis N., Bhalraam U., Reinhold J., Wickramarachchi U., Maart C., Gilbert T., Richardson P., Sulfi S. (2023). Assessment of paclitaxel drug—Coated balloon only angioplasty in STEMI. JACC Cardiovasc. Interv..

[14] Rissanen T.T., Uskela S., Eränen J., Mäntylä P., Olli A., Romppanen H., Siljander A., Pietilä M., Minkkinen M.J., Tervo J. (2019). Drug-coated balloon for treatment of de-novo coronary artery lesions in patients with high bleeding risk (DEBUT): A single-blind, randomised, non-inferiority trial. Lancet.

[15] Corballis N., Merinopoulos I., Bhalraam U., Gunawardena T., Tsampasian V., Natarajan R., Wickramarachchi U., Mohamed M., Clark A., Mamas M.A. (2025). SPARTAN-Norfolk Consortium. A Comparison of a Drug Coated Balloon with Drug Eluting Stent Strategy for Treating Coronary Bifurcation Lesions. Catheter. Cardiovasc. Interv..

[16] Merinopoulos I., Gunawardena T., Corballis N., Bhalraam U., Gilbert T., Maart C., Richardson P., Ryding A., Sarev T., Sawh C. (2022). Paclitaxel drug-coated balloon-only angioplasty for de novo coronary artery disease in elective clinical practice. Clin. Res. Cardiol..

[17] Jeger R.V. (2023). Drug-Coated Balloons in Multivessel Coronary Artery Disease. JACC Cardiovasc. Interv..

