# Supplementary material for: Assessment of Paclitaxel Drug-Coated Balloon-Only Angioplasty for Multivessel Coronary Artery Disease
Source: J Clin Med. 2025 Dec 26;15(1):204. doi: 10.3390/jcm15010204 (PMC12786437; doi:10.3390/jcm15010204)
Supplement: Supplementary file 1 [file jcm-15-00204-s001.zip › jcm-4026682-supplementary.pdf]

Supplementary Materials

Supplementary Table S1. ICD-10 codes used to identify patients' outcomes.

**Acute coronary syndrome**	
STEMI	I210* I211* I212* I213
NSTEACS	I214 I219 I200 (UA)
Re-infarction	I22*
**Stroke**	
Ischaemic stroke	I63*
Transient ischaemic attack	G459, G453
**Major bleeding**	
Subarachnoid haemorrhage	I60*
Intracerebral haemorrhage	I61*
Non-traumatic intracranial bleeding	I62*
Gastrointestinal bleeding	K920, K921, K922
Haemorrhage not elsewhere specified	R58X

Supplementary Table S2. Univariable Cox regression model for MACE.

**MACE** (Univariate)	**N**	**HR (95% CI)**	**p-value**
DES	381	0.99 (0.69 to 1.42)	0.96
Age	381	1.01 (1.00 to 1.03)	0.17
Female	381	1.11 (0.72 to 1.71)	0.63
Presentation	381		
STEMI		—	
NSTEMI		1.04 (0.61 to 1.76)	0.89
Elective		0.55 (0.32 to 0.95)	**0.033**
Left main stem PCI		1.71 (1.13 to 2.59)	**0.012**
Hypercholesterolaemia	381	1.17 (0.80 to 1.72)	0.42
Hypertension	381	1.14 (0.80 to 1.62)	0.48
PVD	381	1.63 (0.80 to 3.34)	0.18
Stroke	381	1.35 (0.66 to 2.77)	0.41
Myocardial infarction	381	1.21 (0.83 to 1.78)	0.33
Previous PCI	381	1.29 (0.88 to 1.88)	0.19
CABG	381	1.85 (1.08 to 3.18)	**0.026**
Heart Failure	381	1.06 (0.39 to 2.86)	0.91
Atrial fibrillation	381	2.42 (1.26 to 4.64)	**0.008**
COPD	381	1.16 (0.56 to 2.37)	0.69
Diabetes	381	1.78 (1.22 to 2.59)	**0.003**
Family history of IHD	381	0.45 (0.25 to 0.80)	**0.006**
Current/Ex Smoker	381	1.08 (0.75 to 1.56)	0.69
GFR	380	1.0 (0.99 to 1.00)	0.12
Heavy calcification	381	1.53 (1.07 to 2.20)	**0.021**
Diffuse disease	381	1.05 (0.71 to 1.54)	0.81
Tortuosity	381	1.34 (0.85 to 2.13)	0.21
Vessel Diameter	381	1.03 (0.70 to 1.50)	0.89
Lesion Length	381	1.00 (0.99 to 1.01)	>0.99

Abbreviations: CI = Confidence Interval, HR = Hazard Ratio

Supplementary Table S3. Univariate Cox regression analysis for propensity score matched cohort.

**MACE** (Univariate)	**N**	**HR (95% CI)**	**p-value**
PCI strategy	306		
DCB-only		—	
DES		1.16 (0.79 to 1.70)	0.46
Age	306	1.01 (0.99 to 1.03)	0.18
Female	306	1.08 (0.66 to 1.77)	0.77
Presentation	306		
STEMI		—	
NSTEMI		0.97 (0.53 to 1.75)	0.91
Elective		0.59 (0.32 to 1.07)	0.084
Left main stem PCI	306	1.48 (0.93 to 2.35)	0.10
Hypercholesterolaemia	306	1.22 (0.80 to 1.88)	0.35
Hypertension	306	1.23 (0.84 to 1.81)	0.28
PVD	306	1.35 (0.63 to 2.91)	0.44
Stroke	306	1.43 (0.69 to 2.94)	0.33
Myocardial infarction	306	1.07 (0.69 to 1.64)	0.76
Previous PCI	306	1.24 (0.82 to 1.87)	0.31
CABG	306	1.79 (1.03 to 3.09)	**0.038**
Heart failure	306	0.82 (0.26 to 2.58)	0.73
Atrial fibrillation	306	2.31 (1.20 to 4.46)	**0.013**
COPD	306	1.08 (0.47 to 2.46)	0.86
Diabetes	306	1.78 (1.18 to 2.68)	**0.006**
Family history of IHD	306	0.49 (0.27 to 0.90)	**0.021**
GFR	306	0.99 (0.99 to 1.00)	0.16
Heavy calcification	306	1.76 (1.20 to 2.60)	**0.004**
Diffuse disease	306	1.11 (0.74 to 1.68)	0.61
Tortuosity	306	1.30 (0.80 to 2.11)	0.30
Vessel diameter	306	1.13 (0.75 to 1.69)	0.57
Lesion length	306	1.00 (0.99 to 1.01)	0.96

Abbreviations: CI = Confidence Interval, HR = Hazard Ratio
